# Study protocol for an open-label, single-arm, mixed methods feasibility study of the MWIQ AI-powered decision support tool for diabetes management in GP practices

**DOI:** 10.1136/bmjopen-2024-097644

**Published:** 2025-10-05

**Authors:** Jane Dickson, Scott G Cunningham, Chris Sainsbury, Martin K Rutter, N Kanumilli, Ewan Pearson, Doogie Brodie, Marryat Stevens, Deborah J Wake, Nicholas Conway

**Affiliations:** 1 Medical School, University of Dundee, Dundee, Angus, UK; 2 University of Birmingham, Birmingham, UK; 3 Division of Diabetes, Endocrinology and Gastroenterology, The University of Manchester, Manchester, UK; 4 Manchester Diabetes Centre, Manchester University NHS Foundation Trust, Manchester, Greater Manchester, UK; 5 Northenden Group Practice, Northenden, UK; 6 My Way Digital Health LTD, Dundee, UK; 7 Usher Institute, The University of Edinburgh, Edinburgh, UK; 8 Ninewells Hospital and Medical School, Dundee, UK

**Keywords:** Diabetes Mellitus, Type 2, Digital Technology, DIABETES & ENDOCRINOLOGY, Clinical Protocols, eHealth, Clinical Decision-Making

## Abstract

**Introduction:**

Diabetes affects ~10% of the world’s population and is rising. Treatment costs in the UK are ~15% of the NHS budget. Diabetes-related complications can be lowered through better evidence-based clinician management and patient self-management. MyWay intelligence quotient (MWIQ) is an electronic platform that will provide clinical decision support around the diagnosis and treatment of patients with diabetes. This study evaluates the safety and clinical performance (clinical appropriateness/applicability, clinical impact and clinical usability) of MWIQ.

**Methods and analysis:**

The system will be implemented in real time in four to seven general practitioner (GP) practices. Clinicians with diabetes expertise will be recruited as validators, who will inspect records to ensure system robustness before use, and up to 14 healthcare professionals will use and evaluate the system.

Quantitative and qualitative analyses will be triangulated to assess the MWIQ system. Assessment of clinical outcomes will be made using pseudonymised routinely collected clinical data, including adherence to quality performance indicators, diabetes diagnosis, diabetes investigations (eg, genetic testing), HbA1c, blood pressure, body mass index, cholesterol and foot risk score for the diabetes population concerned. Clinical and validator participants will also submit a weekly questionnaire, and these, along with interviews, which are scheduled during the testing process, will be analysed to provide data on the utility, safety and usability of the system.

**Ethics and dissemination:**

This study was approved, 08/01/2024, by the North of Scotland Research Ethics Committee (REC), IRAS project ID: 305267, REC, reference 23/NS/0134. The study has gained confidentiality advisory group (CAG) support (reference: 24/CAG/0002), medicines and healthcare products regulatory agency (MHRA) and health research authority (27/08/2024) approvals.

Findings will be reported to (1) The funding body, (2) The participating GP practices, (3) The study PPIE group, (4) The MHRA to support a submission for recognition as a class 2 CE/UKCA marked device, (5) Presented at local, national and international conferences and (6) Disseminated by peer-reviewed publications.

**Trial registration number:**

ISRCTN17422256.

**STRENGTHS AND LIMITATIONS OF THIS STUDY**

- Testing in ‘real-world’ circumstances ensures that clinician participant feedback is representative of system usage within a normal, busy clinical context, making results more generalisable.
- Similarly, the involvement of multiple primary care centres will ensure that any findings are more generalisable to the wider primary care community.
- Using validators to mitigate potential risks strengthens the testing framework.
- The quantitative analysis will be limited to providing descriptive statistics rather than statistical inference. These feasibility data will inform the design of future planned intervention studies.
- Any technical issues that develop during testing may shorten the length of time the system is tested.

## Introduction

 Diabetes affects ~10% of the world’s population and is rising. Treatment costs in the UK are ~15% of the NHS budget; 40% of which is spent on treating diabetes-related complications.^1 2^ The majority of these complications are preventable through better clinician evidence-based management and patient self-management.^2^

Precision medicine approaches through digital clinical decision support systems (CDSS) have been shown to offer safe and effective ways to manage diabetes and are starting to demonstrate improvements in clinical outcomes,^3 4^ including improvements in key parameters such as HbA1c.^5^ These systems are also starting to prove cost-effective.^6^ In addition, the potential for providing patients with information for self-management is high.^7 8^ But as the American diabetes association/European association for the study of diabetes consensus report highlighted, more work is required in this area.^9^

A precision-medicine approach with automated clinical decision support lends itself well to diabetes, where diverse factors are taken into account when managing a condition.^10^ Key areas include precision diagnostics, therapeutics and prognostics.^8^ Diabetes diagnostic sub-typing is complex, with approximately 15% of people incorrectly classified,^11^ potentially leading to inappropriate, higher risk/costly medications (including unnecessary use of insulin injections) and a significant impact on quality of life. Underdiagnosed subtypes include: (1) Genetic types known as maturity onset diabetes of the young, (2) Latent onset autoimmune diabetes of adulthood and (3) Diabetes due to secondary causes. In addition, type 2 diabetes has multiple distinct phenotypes.^11 12^ Drug response is also highly dictated by phenotype and genotype,^13^ yet treatment protocols are not always tailored in this way, which continues the lack of personalisation of results and ineffective medications/polypharmacy.^14^ In addition, most patients are managed in primary care where specialist input may be limited and where complications risk factor management in diabetes is rarely optimised.^15 16^

This study will contribute to the evidence base for AI-driven digital CDSS in use for chronic disease management through the development and testing of MyWay intelligence quotient (MWIQ).

### MyWay intelligence quotient (MWIQ)

MWIQ V.1.1 is an online electronic platform that provides clinical decision support around the diagnosis and treatment of patients with diabetes and will be delivered as part of the MyWay clinical and MyWay diabetes patient-facing platforms. The MWIQ clinician interface tool contains ~70 predictive models, generated through machine learning techniques and developed by MyWay digital health (MWDH). These models will be operationalised to predict glycaemic/metabolic response to type 2 diabetes pharmacological treatments, diagnosis of rare diabetes subtypes and risk of diabetes-related complications. In addition, they will provide a clinical overview of patient populations.

MWIQ machine learning models were developed through validated literature-based models, then the development of meta-models derived from them and the creation of de-novo models.

The validated literature and meta-models development predict diabetes subtype, diabetes acute and a large range of chronic complications, including cardiovascular disease, renal complications, amputation, blindness, hypoglycaemia, hospitalisation, re-hospitalisation, mortality, etc, and response to lifestyle interventions. The drug response prediction uses multi-dimensional time series data to predict the likely response to defined medications (counterfactual conditions) in domains of interest (HbA1c, blood pressure (BP), etc). Predicted outcomes guide individual therapeutic choice.

MWIQ will provide clear guideline-linked evidence-based decision support, particularly helping primary care staff who may lack specialist knowledge, by building on basic rule-based decision support, which has previously been shown to improve compliance with the guidelines by around 3–4 fold.^17^ Another important feature of the MWIQ system is the human factors approach, which is built into the development and research from the start.^18^

**Figure 1:**
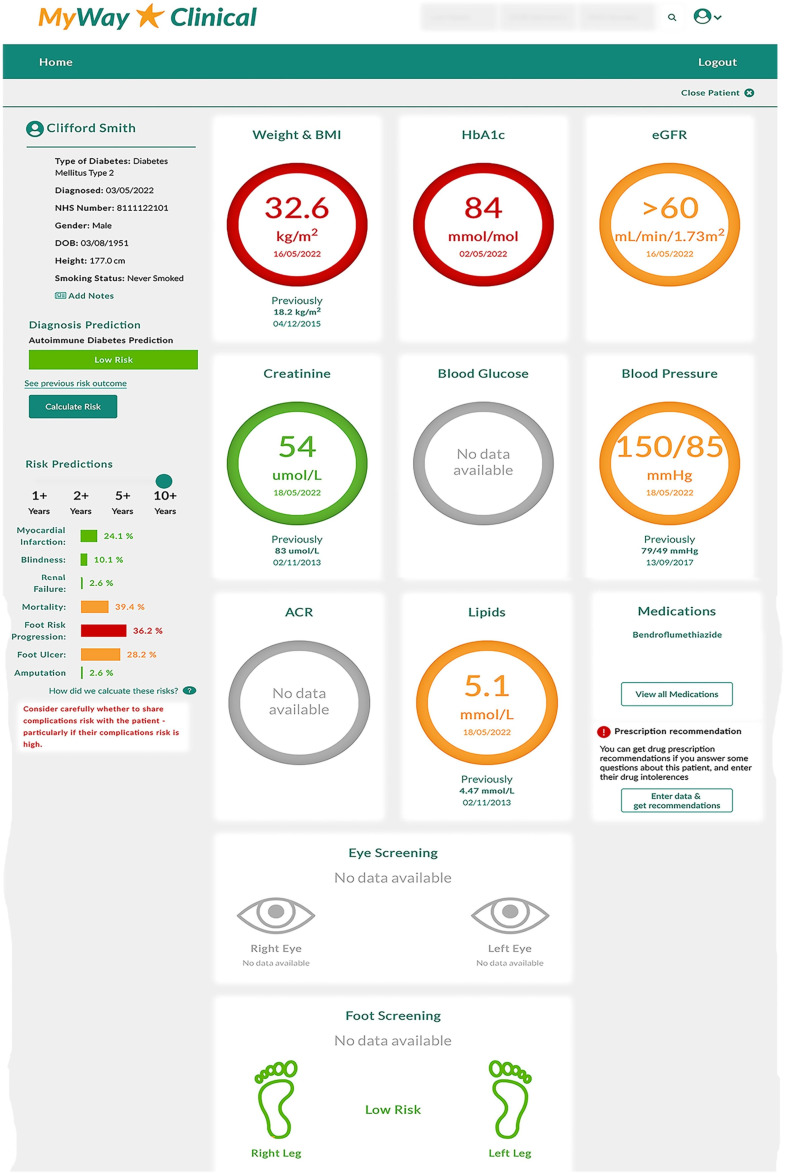
The MyWay IQ clinician’s view for a (fictitious) patient. Includes data pertaining to body mass index (BMI); haemoglobin A1c (HbA1c); estimated glomerular filtration rate (eGFR); albumin to creatinine ratio (ACR).

MWIQ aims to improve adherence to existing guidelines, while providing tailored treatment and diagnostic recommendations (within these existing guidelines) to improve future clinical outcomes (figures1  2). MWIQ is not intended to replace the care provided by licensed healthcare professionals (HCPs), involving prescribing, diagnosis or treatment. The system is supportive and advisory. Any treatment decision ultimately requires clinical judgement, taking the full clinical scenario into consideration.

**Figure 2:**
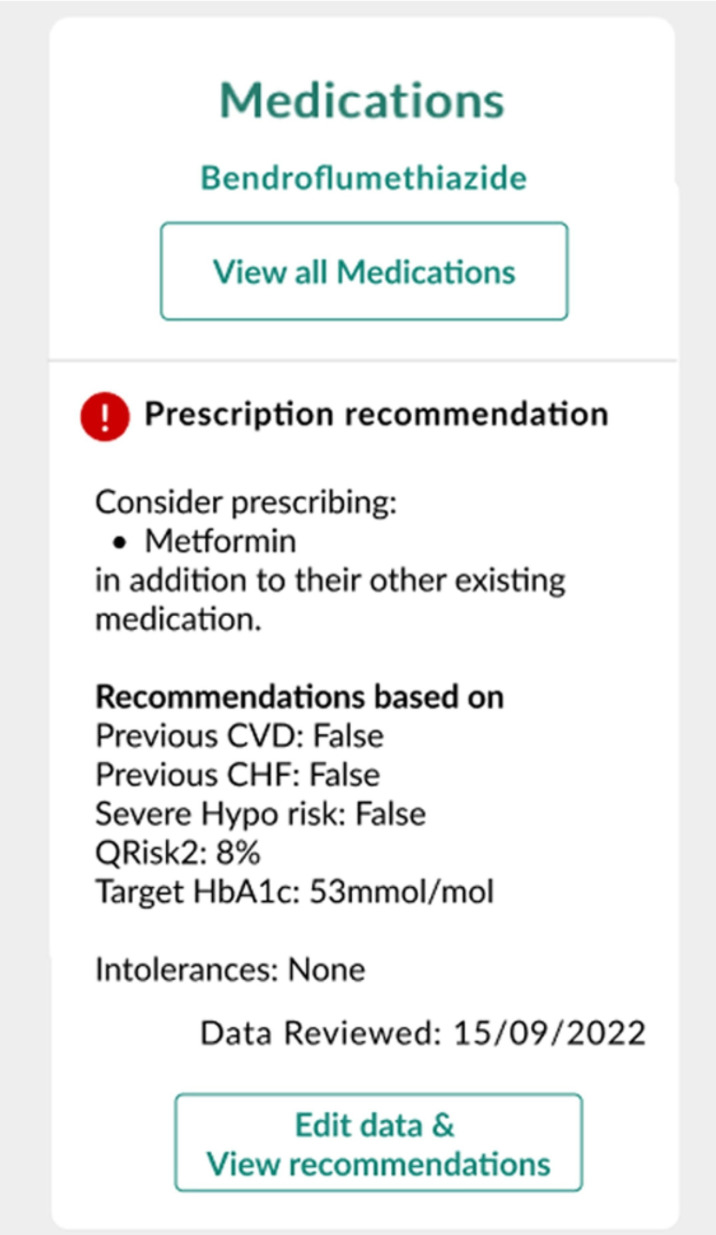
MyWay IQ example of a (fictitious) medication recommendations panel. Takes into account presence or absence of cardiovascular disease (CVD); congestive heart failure (CHF); and cardiovascular risk score (QRisk2).

## Study aim

The study will use real-world environments to assess the clinical safety and performance of MWIQ. The primary objective is to iteratively test, identify and mitigate risks in the MWIQ system through a systematic evaluation of the platform’s safety in clinical settings. The secondary objectives are to evaluate the clinical performance of MWIQ measured in three areas: clinical appropriateness (applicability), clinical impact and clinical usability. A successful outcome will support a submission to MHRA for recognition as a class 2 CE/UKCA-marked device.

### Research question

Is the use of MWIQ safe, appropriate, usable and effective when used by primary care HCPs involved in the care of people with diabetes, in comparison to usual care?

## Methods

### Study design

This study is an open-label, single-arm, mixed-methods feasibility testing of a support and decision-aid clinician-facing platform. Real-world testing will take place within the integrated care board, NHS Greater Manchester (the single site), with four to seven general practitioner (GP) surgeries participating across this large multicultural British city. The study contacted all practices within this area, and five practices responded positively. The study started recruitment for clinical participants on 28/08/2024. Live clinical testing started in March 2025, and the end of the study is 08/09/2025. Each participating practice serves a population of approximately 285 diabetes patients. Therefore, within the study period, it is anticipated that ~665 people with diabetes will attend their primary care practice for a diabetes check.

We will recruit one or two clinician participants from each of 5 GP practices, for a maximum of 10, to ensure the device is used in a variety of diabetes-related clinical settings and comprises a broad range of age groups, ethnicities and medical needs. Clinician participants will be members of the multidisciplinary team (GPs, specialist registered nurses (RNs) and advanced nurse prescribers who regularly see people with diabetes as part of their day-to-day work. They will provide a real-world perspective on how well the system integrates within the normal clinical workflow and provide feedback on how well the system performs. Sample size is calculated to withstand a small number of dropouts, while being large enough to draw out user views with different qualitative and quantitative methods and detect any safety issues across the long term. Each clinician participant will be invited to interview across the testing period (up to a maximum of 10 interviews). This is based on the narrow and focused scope of the interviews, collecting rich interview data by continuous interviewing throughout the study and the variety of HCPs involved in diabetes care in the primary sector and the anticipated number to reach data saturation for this type of study.

In addition, four validator participants will be recruited to review the patient data for safety issues. The validator participants will review the case records of all people with diabetes registered within the participating practice in advance of (and following) a scheduled diabetes review in the participating practice. Validation will consider the safety, applicability and usefulness of the system outputs. Any safety concerns will be flagged and investigated, and clinical participants will be advised not to access the patient record until the issue is resolved. Confidential advisory group approval has been granted to enable clinical validators to access relevant patient records in the participating clinics.

### Patient and public involvement

Patients are not participants in this study. Patients attending appointments in the participating GP practices will be informed of the study by letter/email/text message. They can then opt out of having their data used in the study by informing their GP practice or the research team.

A patient public involvement (PPI) group of five members was recruited via VOCAL, a local Manchester organisation, which connects people with research studies. The PPI was convened during the initial planning phase. The members of the group comprise two men and three women who reside in the NHS Greater Manchester health board. They range in age, physical ability, ethnicity and diabetes type. The PPI group reviewed the clinician-facing system during interface development, and their concerns and suggestions were incorporated into the system and protocol design. The PPI group also reviewed the patient-facing letter and co-designed the poster and leaflet. Finally, the group will be asked to co-design and review study dissemination materials. In addition, one patient representative serves on the study board.

### Inclusion

All participants will be 18 years or over, able to provide informed consent and not be employed by MWDH, involved in design and development or an employee or paid consultant for any marketing research company, national regulatory agency or medical device manufacturer.

Clinician participants will be qualified HCPs (GP or RN) involved in the care of people with diabetes within NHS Greater Manchester.

Validator participants will be diabetes specialists with appropriate medical qualifications (eg, general medical council certificate of completion of training).

### Exclusion

Clinicians who do not meet the inclusion criteria.

### Data collection

The study objectives will be assessed through a range of triangulated qualitative and quantitative methods. First, pseudonymised routinely collected clinical data will be collected for quantitative analysis. This will provide an overview of the diabetes population served by the clinician participants (see tables1  2).

**Table 1:** Primary outcome linked to methods for MyWay intelligence quotient testing

Primary outcomes linked to methods for MWIQ testing			
**PRIMARY OUTCOME**	**DESCRIPTION**	**METHODS**	**MEASURES TO BE EMPLOYED**
System safety	Identify and mitigate risks in the MWIQ system through a systematic evaluation of the platform’s safety in clinical settings	Weekly questionnaires End-of-study questionnaires Unsolicited issues highlighted by participants	Number of risks identified by patient-level questionnaires (interim and cumulative total), expressed as a percentage (and binomial proportion CI) of number of the patient records reviewed Clinical safety score recorded within the weekly questionnaire for each of the MWIQ features (diabetes subtype predictor, diabetes drug response predictor and diabetes complications predictor), as well as overall experience. This score will be recorded via a 5-point Likert scale, and the distribution of scores will be expressed as a percentage of responses received Clinical plausibility score recorded within the weekly questionnaire for each of the MWIQ features (diabetes subtype predictor, diabetes drug response predictor and diabetes complications predictor) as well as overall experience. The score will be recorded via a 5-point Likert scale, and the distribution of scores will be expressed as a percentage of responses received Free-text comments will be analysed for emergent themes

MWIQ, MyWay intelligence quotient.

**Table 2:** Secondary outcomes and the method of testing for MyWay intelligence quotient testing

Secondary outcomes linked to methods for MWIQ testing		
**SECONDARY OUTCOMES**	**METHODS**	**MEASURES TO BE EMPLOYED**
Clinical impact	Routinely collected clinical data System navigation data Weekly questionnaire data	Number of patients with the following care processes recorded within the past 12 months: HbA1c, blood pressure (BP), serum cholesterol, serum creatinine, urinary albumin, foot screening, body mass index and smoking status. Each will be expressed as a percentage of the total number of people with diabetes seen by participants within the study period (and binomial proportion CI). Analysis will be stratified in accordance with diabetes type, age, gender, ethnicity and socioeconomic status (derived via the English index of multiple deprivation score) Number of patients meeting the following treatment targets: HbA1c <58 mmol/mol, BP=<140/80 mm Hg, Cholesterol <5 mmol/L, as well as the number meeting all three targets. Each will be expressed as a percentage of the total number of people with diabetes seen by participants within the study period (and binomial proportion CI) Number of patients with reclassified diabetes diagnosis, expressed as a percentage of the total number of people with diabetes seen by participants within the study period (and binomial proportion CI) and calculated on a monthly basis to demonstrate secular trends (if any) Number of patients with a modification in number of/dose/type of diabetes-related prescriptions (ie, insulin, metformin, DPP-4 inhibitors, pioglitazone, sulphonylurea and/or SLGT2-inhibitors) expressed as a percentage of the total number of people with diabetes seen by participants within the study period, calculated on a monthly basis to demonstrate secular trends (if any). Subjective assessment of system impact—recorded via a 5-point Likert scale. The distribution of scores will be expressed as a percentage of responses received Free-text comments submitted via questionnaire were analysed for emergent themes Number of participant interactions with the system, time spent within the system (cumulative total and per patient)
System appropriateness/applicability	Weekly questionnaires Semi-structured interviews	Clinical usefulness score recorded within weekly questionnaire for each of the MWIQ features (diabetes subtype predictor, diabetes drug response predictor and diabetes complications predictor) as well as overall experience. The score will be recorded via a 5-point Likert scale, and the distribution of scores will be expressed as a percentage of responses received Free-text comments will be analysed for emergent themes
System usability	Weekly system usability scale questionnaires End of study system Usability scale questionnaires Semi-structured interviews	Overall ease of use, recorded via a 5-point Likert scale. The distribution of scores will be expressed as a percentage of responses received Free-text comments and semi-structured interviews will be analysed for emergent themes

MWIQ, MyWay intelligence quotient.

### Quantitative data collection

Quantitative data will be extracted from the MyWay server and pseudonymised prior to secure file transfer to the research team. These pseudonymised data will provide an overview of the diabetes population served by the clinician participants. Data collected will include adherence to quality performance indicators, diabetes diagnosis (ie, diabetes type) and diabetes investigations (eg, genetic testing). Other routinely collected clinical data of interest include HbA1c, BP, body mass index, cholesterol and foot risk score for the diabetes population concerned. The time spent by participants within the MWIQ system will also be evaluated.

Validation questionnaires for each patient include whether the record was reviewed pre-consultation or post-consultation and any comments on the validation (eg, if the medication recommendation is what they expect). Weekly ease of use questionnaires, completed by each clinical participant, ask how easy completing tasks within each tool (diagnostic subtype, medication recommendation and the complications risk) is and for any further comments.

End-of-study questionnaires will be issued to each clinical participant and will ask for a summary of their experience over the testing period using a Likert scale to assess the safety of the diagnostic sub-type, medication recommendation and complications risk tools, clinical appropriateness, clinical flow, difficulty in using the system and any recommendations. This questionnaire will incorporate the system usability scale.^19 20^

### Qualitative data collection

Qualitative data will be gathered from free-text sections of the weekly questionnaires. Clinician participants will be invited for a semi-structured online interview to discuss the complexity of using the system in situ. Interviews will be 30–45 mins long and explore questions about what worked well and not-so-well in practice; which features were most useful, consultation flow when using the system, sharing screens with patients and using the system collaboratively with colleagues, safety, trust and what would make the system better. We will conduct interviews regularly throughout the testing phase and ask to interview all the participating clinician participants twice during this time, if possible.

### Data management

Data is managed by the MWDH data management platform, a Microsoft Azure cloud-based platform developed as the backbone to all MWDH data services.

The data originates in NHS GP systems using several agreed standard formats such as encrypted CSV files from software platforms used in primary care, eg, EMIS (formerly known as Egton Medical Information Systems), which are sent using secure file transfer protocol, and data in JSON via a REST API. They are transformed during import into the MyWay internal standard to enable immediate AI/ML processing without further reformatting.

Research data will be stored on password-protected computers in electronic databases. Participants will be given participant numbers for anonymisation, and informed consent forms will be stored separately from research data. Research data will be kept for 7 years after the study ends.

### Analysis

Run charts will be used to record usability and safety issues identified within questionnaire returns and will be collated and reviewed weekly. Reports will be supplied to the MDMW clinical and technical teams with the issues which require review. Iterative improvements to the system will be based on a review of these comments and suggestions.

The transcribed interviews and free text boxes of the questionnaires will be analysed in NVivo software, and the 8-dimensional sociotechnical model^21 22^ that guided the design of the interview guide will be used. Emergent themes will be analysed with reference to the overall analytical framework to develop a detailed understanding of how clinician participants understood the clinical system, the process of normalising its use in daily practice and prompt for areas for intervention optimisation and refinement.

Overall analysis will be informed by the MRC guidelines on developing and evaluating complex interventions^23 24^ and will consist of descriptive statistical analysis of the quantitative data^25 26^ triangulated with the qualitative analysis to produce a rigorous analysis. This will result in a descriptive comparison between the use of participant-reported, researcher-observed and routinely collated quantitative data. The findings will also guide any refinement of the MWIQ system to accomplish the stated objectives.

## Testing protocol

Clinical participants attend an information session and provide written informed consent to the study team before being trained in the use of the system. No additional software is required to access the MWIQ system, and the validators and clinician participants do not require any additional expertise to operate the system. Validators and clinician participants are given an online link before they start testing. Once signed into the system, they can review a patient’s record. Only clinician participants can input any additional clinical data, for example, test results or changes in smoking status, to generate outputs from the system. The system will not generate an output if there is insufficient clinical data input.

### Ensuring safety during testing

Prior to the system going live within primary care, Validator participants will review real-world patient data in the context of a ‘simulated clinic’. Feedback will be sought on system performance, usability and any identifiable potential risks. The results of this ‘simulated clinic’ testing will be provided to and reviewed by a data and safety monitoring board (DSMB), which is composed of three diabetes experts. They will make a go/no-go decision on proceeding with the study. The DSMB will meet monthly for the duration of the study to review emergent data. Ongoing data collection is subject to their review.

**Figure 3:**
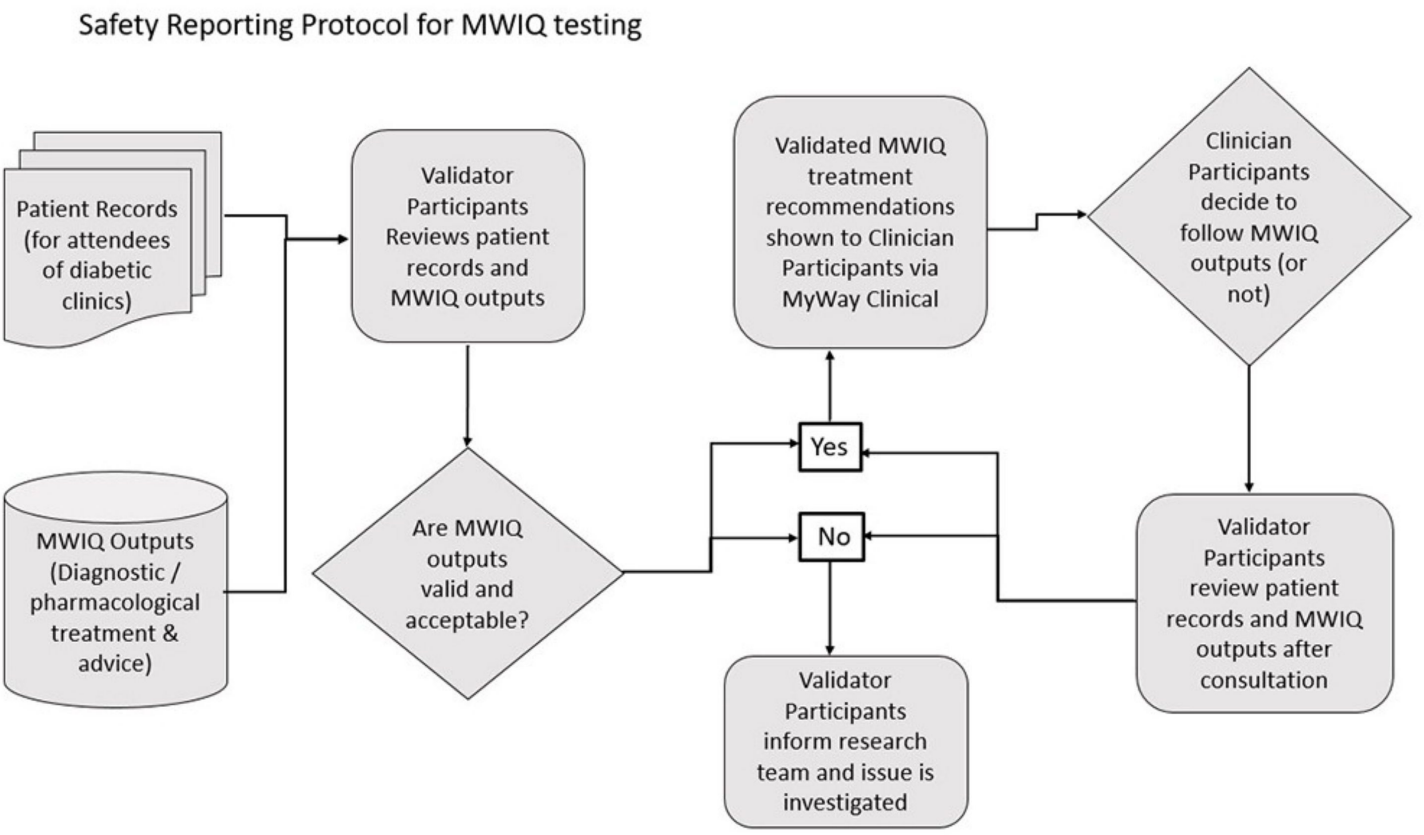
Safety reporting protocol for MWIQ testing. MWIQ, MyWay intelligence quotient

Safety will be ensured and evaluated by:

- The clinician participants will be trained to use the MW IQ system before testing commences.
- Clinicians will exercise their usual clinical judgement with each patient.
- Validator participants will review the patient data and submit an assessment report for each person seen by the clinician participants pre-consultation and postconsultation (figure 3).
- Clinician participants provide review through weekly questionnaires and interviews.
- A yellow card-type system will be in operation for reporting urgent safety issues.
- Any flagged safety issues and their reports will be collated and reviewed by the MYIQ clinical team. They will then be submitted to the study DSMB).
- Pass/fail criteria to be applied to the result of the clinical investigation, and the DSMB will consider safety data before determining whether to proceed.

### Previous studies

Low-risk features of MWIQ (formerly known as MyDiabetes IQ) (Class 1 CE-marked device) have been studied through iterative co-design involving initial focus groups, think-aloud user testing of prototypes and task-based testing by clinicians. Qualitative feedback from users has resulted in iterations made to the user interface and functionality.^24^

## Discussion

The submission requirements for each ethical board (REC Committee, MHRA and CAG) vary, which resulted in a substantial administrative burden for both the research team and the sponsor. It is recommended that those embarking on similar projects include sufficient time to complete the regulatory requirements prior to the planned start date for recruitment, to ensure that project milestones are achievable.

## Anticipated results and potential clinical impacts

Study findings will inform the design and ongoing improvement of the MWIQ clinical system and will be published in peer-reviewed journals. The study will also support a submission to MHRA for recognition as a class 2 CE/UKCA-marked device.

## Ethics and dissemination

This study, *MyWay IQ (MWIQ)—safety and efficacy testing of a diagnosis and precision medicine tool for diabetes management*, with the acronym *AI award MWIQ*, Protocol V.5 dated 24/10/2024, has been approved, 08/01/2024, by the North of Scotland Research Ethics Committee (REC), IRAS project ID: 305267, REC, reference 23/NS/0134. The study also sought and gained confidentiality advisory group (CAG) support (reference: 24/CAG/0002), medicines and healthcare products regulatory agency (MHRA) and health research authority (27/08/2024) approvals. The trial is registered with ISRCTN, as ‘MWIQ—safety and efficacy testing of a diagnosis and precision medicine tool for diabetes management’, ISRCTN17422256, dated 27/01/2022.

The DSMB is composed of three experts in diabetes care who are independent from the sponsor and have no competing interests. The study sponsor is My Way digital health. 163 Bath Street, Glasgow, Scotland, G2 4SQ. The sponsors co-developed the study design and have received ongoing reports of the results so that they can iteratively improve the system, clinically and technically. They do not have input into data collection, management, analysis and interpretation. They will be co-authors in publications. The sponsor has developed an audit protocol for trial conduct, and this is independent from the study team.

The findings from the real-world study will be disseminated to (1) the funding body, (2) the participating GP Ppractices, (3) the study PPIE group, (4) the MHRA to support a submission for recognition as a class 2 CE/UKCA marked device, (5) presented at local, national and international conferences and (6) disseminated by peer-reviewed publications.

Study investigators have the right to publish orally or in writing the results of the study. However, sensitive information concerning the AI intervention and its code, patent applications, processes, scientific data or other pertinent information is confidential and remains the property of My Way digital health.
