# Supplementary material for: Study protocol for an open-label, single-arm, mixed methods feasibility study of the MWIQ AI-powered decision support tool for diabetes management in GP practices
Source: BMJ Open. 2025 Oct 5;15(10):e097644. doi: 10.1136/bmjopen-2024-097644 (PMC12506132; doi:10.1136/bmjopen-2024-097644)
Supplement: Supplementary file 1 [file bmjopen-2024-097644.reviewer_comments.pdf]

PEER REVIEW HISTORY

BMJ Open publishes all reviews undertaken for accepted manuscripts. Reviewers are asked to complete a checklist review form and are provided with free text boxes to elaborate on their assessment. These free text comments are reproduced below.

ARTICLE DETAILS

Title (Provisional)

Study Protocol for an open-label, single-arm, mixed methods feasibility study of the MWIQ AI powered decision support tool for diabetes management in GP Practices

Authors

Dickson, Jane; Cunningham, Scott G; Sainsbury, Chris; Rutter, Martin K; Kanumilli, N; Pearson, Ewan; Brodie, Doogie; Stevens, Marryat; Wake, Deborah; Conway, Nicholas

---

VERSION 1 - REVIEW

**Reviewer**	**1**
**Name**	**Kohlenberg, Jacob**
**Affiliation**	**University of Minnesota**
**Date**	**07-Mar-2025**
**COI**	**None**

This manuscript describes the protocol for a study to evaluate the safety and clinical performance of MyWay Intelligence Quotient (MWIQ), which is an electronic platform that will provide clinical decision support around the diagnosis and treatment of patients with diabetes. This is an impactful project and the manuscript is well written. This manuscript would benefit from some clarifications, as detailed below.

Major comments:

-Background Page 4 lines 3-8: description of the MWIQ clinical interface tool would benefit from additional detail, e.g., which models will be operationalized, how exactly will they be implemented… Suggest providing several examples of how the tool will be used – e.g., present a patient vignette and convey exactly how this tool provides information/recommendations on diagnosis, diabetes-related complication risk, and treatment.

-Methods Page 4: Seven to 14 healthcare professionals will be given access to the system for a period of up to 26 weeks. How many patients is the MWIQ clinical interface tool anticipated to be applied to over the 26 weeks?

-Data collection Pages 5-7: Suggest some clarifications here. 1) include additional details regarding the questionnaires. 2) Provide additional details or citations for the Clinical safety score, Clinical plausibility score, System impact (subjective assessment using a Likert scale), Clinical usefulness score, and Overall ease of use survey. 3) Provide additional details regarding the content of the semi-structured interviews.

Minor comments:

- Background Page 3 lines 22-23: the systematic reviews that are cited were published between 2011 – 2015. Given the number of diabetes related digital tools and apps that have been developed over the past decade, would consider citing more recently published studies/reviews as well.

-Background Page 3 line 44: “around 30% of people are incorrectly classified” – suggest providing a citation for this. If this is citation #15, the citation should likely be included for this statement. Citation #15 was published in 2012, consider a more recent reference given the increase in availability of islet autoantibody testing, genetic testing, etc., over the past decade.

-Methods Page 4 lines 46-47: line 46 states it is a single-site study, line 47 states “real-world testing will take place in four to seven GP surgeries”, suggest clarifying this.

-Suggest including a sample image of the how the MWIQ tool visually appears to the user.

-Secondary outcomes table: “have had their diabetes-related prescriptions modified” … clarify, modified in what manner? Change in dose, medication type, etc.

-References section – seems to be an issue with missing details for reference numbers two and nine

---

VERSION 1 - AUTHOR RESPONSE

Issue highlighted in the review	Page number	Author’s comment
Background Page 4 lines 3-8: description of the MWIQ clinical interface tool would benefit from additional detail, e.g., which models will be operationalized, how exactly will they be implemented… Suggest providing several examples of how the tool will be used – e.g., present a patient vignette and convey exactly how this tool provides information/recommendations on diagnosis, diabetes-related complication risk, and treatment.	2-5	We were working under the BMJ Open’s instruction of keeping the manuscript as succinct as possible but clearly neglected to include valuable information the reader requires.<br>More detail about the model development is now included, as is a description of how the tool will be used. New figures have been added.

Methods Page 4: Seven to 14 healthcare professionals will be given access to the system for a period of up to 26 weeks. How many patients is the MWIQ clinical interface tool anticipated to be applied to over the 26 weeks?	6	Added to paragraph on methods and sample size
Data collection Pages 5-7: Suggest some clarifications here. 1) include additional details regarding the questionnaires. 2) Provide additional details or citations for the Clinical safety score, Clinical plausibility score, System impact (subjective assessment using a Likert scale), Clinical usefulness score, and Overall ease of use survey. 3) Provide additional details regarding the content of the semi-structured interviews.	6-9	1) questionnaire details added<br>2) added into table on primary outcomes linked to methods and into table on Secondary outcomes<br>3) interviews - sample questions have been added
Background Page 3 lines 22-23: the systematic reviews that are cited were published between 2011 – 2015. Given the number of diabetes related digital tools and apps that have been developed over the past decade, would consider citing more recently published studies/reviews as well.	2	The background section has been revised in light of the comments.
Background Page 3 line 44: "around 30% of people are incorrectly classified" – suggest providing a citation for this. If this is citation #15, the citation should likely be included for this statement. Citation #15 was published in 2012, consider a more recent reference given the increase in availability of islet autoantibody testing, genetic testing, etc., over the past decade.	2	A more recent reference has been included, and it is encouraging to see that the incidence of misdiagnosis has fallen.
Methods Page 4 lines 46-47: line 46 states it is a single-site study, line 47 states "real-world testing will take place in four to seven GP surgeries", suggest clarifying this.	5	Single site study refers to one single NHS board (not the number of GP Practices) – however, to avoid confusion this has been removed from the study description.
Suggest including a sample image of the how the MWIQ tool visually appears to the user.	3, 4	Great idea, thanks, this has been included.

Secondary outcomes table: “have had their diabetes-related prescriptions modified” … clarify, modified in what manner? Change in dose, medication type, etc.	7-8	This has been further expanded.
References section – seems to be an issue with missing details for reference numbers two and nine	13-15	References have been updated and the relevant details added.
Additional change –update to the end of study	6	The study received an extension to allow testing to continue for as long as possible. End date is now September 2025
